# The EUPEMEN (EUropean PErioperative MEdical Networking) Protocol for Bowel Obstruction: Recommendations for Perioperative Care

**DOI:** 10.3390/jcm12134185

**Published:** 2023-06-21

**Authors:** Orestis Ioannidis, Jose M. Ramirez, Javier Martínez Ubieto, Carlo V. Feo, Antonio Arroyo, Petr Kocián, Luis Sánchez-Guillén, Ana Pascual Bellosta, Adam Whitley, Alejandro Bona Enguita, Marta Teresa, Elissavet Anestiadou

**Affiliations:** 1 Fourth Department of Surgery, Medical School, Faculty of Health Sciences, Aristotle University of Thessaloniki, General Hospital “George Papanikolaou”, 57010 Thessaloniki, Greece; 2 Institute for Health Research Aragón, 50009 Zaragoza, Spain; jramirez@unizar.es (J.M.R.); jmtezubieto@hotmail.com (J.M.U.); anapascual689@gmail.com (A.P.B.); mteresa@iisaragon.es (M.T.); 3 Department of Surgery, Faculty of Medicine, University of Zaragoza, 50009 Zaragoza, Spain; 4 Department of Anesthesia, Resuscitation and Pain Therapy, Miguel Servet University Hospital, 50009 Zaragoza, Spain; 5 Department of Surgery, Azienda Unità Sanitaria Locale Ferrara—University of Ferrara, 44121 Ferrara, Italy; carlo.feo@unife.it; 6 Department of Surgery, Universidad Miguel Hernández Elche, Hospital General Universitario Elche, 03203 Elche, Spain; arroyocir@hotmail.com (A.A.); drsanchezguillen@gmail.com (L.S.-G.); 7 Department of Surgery, Second Faculty of Medicine, Charles University and Motol University Hospital, 150 06 Prague, Czech Republic; kocian.cz@gmail.com; 8 Department of Surgery, University Hospital Kralovske Vinohrady, 100 34 Prague, Czech Republic; whitley.adam@gmail.com; 9 Grupo Español de Rehabilitación Multimodal, 50009 Zaragoza, Spain; secretariagerm@gmail.com

**Keywords:** bowel obstruction, perioperative care, care program, surgical rehabilitation, EUPEMEN project

## Abstract

Mechanical bowel obstruction is a common symptom for admission to emergency services, diagnosed annually in more than 300,000 patients in the States, from whom 51% will undergo emergency laparotomy. This condition is associated with serious morbidity and mortality, but it also causes a high financial burden due to long hospital stay. The EUPEMEN project aims to incorporate the expertise and clinical experience of national clinical specialists into development of perioperative rehabilitation protocols. Providing special recommendations for all aspects of patient perioperative care and the participation of diverse specialists, the EUPEMEN protocol for bowel obstruction, as presented in the current paper, aims to provide faster postoperative recovery and reduce length of hospital stay, postoperative morbidity and mortality rate.

## 1. Introduction

While surgery is indicated to for the treatment or palliation of various diseases, in many cases, surgery leads to adverse effects that affect daily living by impairing quality of life and increase the cost of health system because of longer hospitalization time of the patient The goal of the multimodal surgical rehabilitation or enhanced recovery after surgery is the application of a series of perioperative procedure measures and strategies aimed at patients who are going to undergo a surgical procedure with the objective of reducing secondary stress caused by the surgical intervention and thus achieve enhanced recovery of the patient and decrease complications and mortality [1,2]. These care programs are based on scientific evidence, encompass all aspects of patient care, and require multidisciplinary management, with the participation of diverse specialists. Starting at the diagnosis, their aim is to recognize patients’ individual needs, to optimize their treatment before, during and after surgery [1,2].

In order to implement these programs at hospitals in Europe, 5 partners with health and university profile of 4 different EU countries have created the EUPEMEN project. The objective is to prepare a guideline with the protocols to be implemented by the multidisciplinary specialists involved. The Eupemen project has been carried out by Fundación Instituto de Investigación Sanitaria Aragón-IISA as coordinator and Azienda Unità Sanitaria Locale Ferrara—AUSLFE, Univerzita Karlova—CUNI, Universidad Miguel Hernández de Elche—UMH and “G. Papanikolaou—GPAP” General Hospital of Thessaloniki as partners. The main objective of the EUPEMEN project is to create and disseminate protocols for multimodal surgical rehabilitation based on the experience and previous knowledge of the five partners belonging to the health field and higher education. The technical activities of the project included the preparation of the EUPEMEN Multimodal Rehabilitation manual with the protocols of 6 different modules: Bariatric Surgery, Oesophageal Surgery, Gastric Cancer Surgery, Colon Surgery, Hepatobiliary Surgery, and Urgent abdominal surgery, including appendectomy and small bowel obstruction (SBO).

## 2. Bowel Obstruction

Bowel obstruction is one of the most common surgical emergencies worldwide and accounts for about 15% of cases requiring admission for abdominal pain and constitute about 20% of acute surgical cases [3,4]. SBO accounts for the majority of cases with a percentage of about 75–80% while the rest 20–25% are caused by large bowel obstruction [5].

SBO has been recognized as a medical emergency from the ancient years as descriptions of the disease date back even in the era of ancient Egypt and ancient Greece [6,7]. It is a quiet common emergency medical issue as about 2–4% of abdominal pain cases seen in the emergency department and a percentage as high as 12–16% of surgical admissions are due to SBO [6,8]. In developed countries the most common cause of SBO are adhesions from previous abdominal surgery accounting for 65–78% of the cases, while less common but still not so rare causes are hernias and neoplasms accounting for 10% and 5% respectively [6,9,10]. While the classical dogma of surgery “never let the sun set on a small bowel obstruction” has been challenged the last years and there has been increased focus on successful nonoperative management including the gastrografin challenge [8,9] surgery remains a great part of SBO management and SBO accounts for 20% of all emergency surgical procedures and more than 300,000 operations are performed annually for SBO in the USA [6,11].

For the less common large bowel obstruction, the main cause is neoplasm which account for about 50–60%, with volvulus found in 10–20% and diverticula in another 10–20% [3,4,5]. Contrary to SBO, most cases of large bowel obstruction will require surgical treatment as the majority of cases won’t resolve by observation in combination with medical treatment [3,4,5].

## 3. Enhanced Recovery after Surgery (ERAS)

Enhanced Recovery After Surgery (ERAS) is a modern approach to perioperative care of surgical patients that aims to reduce surgical stress and thus improve recovery of patients [1,2]. Enhanced recovery protocols aim to ensure that patients are in the best possible conditions before surgery, receive the most optimal surgery and anaesthesia and postoperative care [1,2]. Implementation of enhanced recovery protocols relies on the close collaboration of all specialists participating in the perioperative process, as well as of the actual patients and their relatives [1,2]. The concept of improving recovery after surgery was introduced in the 1990s by Professor Henrik Kehlet [1]. The original protocols were written for colorectal surgery [12,13]. The protocols have since been shown in several randomized clinical trials and meta-analyses to reduce postoperative complications, length of hospitalization, improve clinical recovery parameters and to reduce hospital costs [14,15,16,17,18,19]. Enhanced recovery protocols have now been developed for a wide range of surgical fields, including foregut surgery, pancreatobiliary surgery, gynecological surgery, and urology [14,15,16,17,18,19]. In 2015, in a further attempt to improve postoperative outcomes, the Intensified Recovery in Abdominal Surgery (Via RICA) protocol was developed and published [20]. Via RICA is a detailed enhanced recovery protocol for abdominal surgery based on interdisciplinary consensus. An update for the Via RICA has since been developed, which includes several other surgical disciplines other than abdominal [21] (https://eupemen.eu/wp-content/uploads/2022/10/Eupemen-Protocol-Bowel-Obstruction.pdf, accessed on 24 April 2023). The articles chosen for review and on which the protocol was based are the same articles there were used to develop the via RICA protocol.

## 4. The EUPEMEN Bowel Obstruction Protocol (Figure 1)

### 4.1. Preoperative Phase

In the preoperative phase of the management of a patient with SBO, which is performed by the surgeon and the anaesthesiologist, it is important to complete the following routine preoperative assessment that includes physical examination, abdominal X-ray and full blood laboratory analysis including C-reactive protein (CRP).

Also, clinical scoring systems should be implemented for the assessment of elderly patients. Fragility scores should be used such as the Modified Frailty Index and VIG Express and the Beers criteria should be reviewed for preventing delirium in adults over 65 years old. Despite the fact that there is no perfect score, any score is better than none [22,23,24,25,26].

Normothermia should be ensured preoperatively in all patients but especially in frail patients by using heat blankets [27,28,29,30]

One of the most crucial issues is perioperative glycaemic control. Specifically, for diabetic patients the local hospital protocols for diabetics undergoing surgery should be used while in patients at risk of developing insulin resistance (obese and elderly patients) and in surgeries lasting more than one hour, blood glucose levels higher than 180 mg/dL should be avoided [31,32,33,34,35].

Contrary to the protocols in non-emergency surgery nasogastric tube placement is recommended in all cases, while urinary catheterization should be avoided and be used only if necessary.

Perioperative care bundles to prevent surgical site infections are highly recommended and antibiotic prophylaxis should be given in all cases and the type of antibiotics should be chosen according to the local hospital policy [36,37,38,39].

Despite the emergent character of the procedure informed consent is required and the patient should be fully informed of the planned procedure and its potential complications, as it decreases hospital stay and allows early discharge. Competent patients should give signed informed consent [12,14,40,41].

### 4.2. Intraoperative Phase

In the preoperative phase of the management of a patient with SBO, which is performed by the surgeon, the anaesthesiologist and the nurse the WHO surgical checklist should be used as it increases patient safety [42,43,44,45].

Regarding the anaesthesiologist approach the intraoperative elements of the EUPEMEN protocol are routine intraoperative monitoring, which should include non-invasive blood pressure measurement, electrocardiogram with 5 leads (V5 and DII recommended), fraction of inspired oxygen (FiO_2_), pulse oximetry (with % O_2_ Saturation), capnography (EtCO_2_) [46,47], central temperature [48,49,50], intraoperative blood glucose and fluid therapy balance, rapid sequence induction for anaesthesia and no face mask ventilation in order to reduce aspiration of gastric contents, perioperative oxygenation with a fraction of inspired oxygen between 0.6 and 0.8. Moreover, goal-directed fluid therapy using non-invasive hemodynamic monitoring systems should be used. If such systems are not available, balanced solutions should be given continuously according to the surgical approach: 3–5 mL/kg/h for laparoscopy and 5–7 mL/kg/h for laparotomy [51,52,53,54]. Also, epidural analgesia should be used in open surgery [55,56] and prophylaxis of postoperative nausea and vomiting should be done by the administration of antiemetic therapy according to the Apfel score [57,58,59].

Regarding the surgical elements of the intraoperative component of the EUPEMEN protocol for SBO minimally invasive approaches should only be used in highly selected cases according to the experience of the surgeon. In most cases open surgery should be preferred and abdominal drains should be avoided as much as possible [14,40,60,61,62,63,64,65]. Furthermore, urinary catheterization should be avoided and be used only if necessary [66,67,68].

In the concept of the multidisciplinary management of those patients it is the responsibility of the whole team to achieve perioperative glycaemic control. For diabetic patients’ local hospital protocols for diabetics undergoing surgery should be used while in patients at risk of developing insulin resistance (obese and elderly patients) and in surgeries lasting more than one hour, blood glucose levels higher than 180 mg/dL must be avoided [69,70,71]. Normothermia should be maintained throughout the procedure by the use of thermal blankets and heated fluids [27,28,30,49,72,73,74,75] and thromboembolic prophylaxis consisting of compression stockings or intermittent compression and low-molecular weight heparin should be given according to the local hospital policy [76,77,78,79,80]. Last, but not least perioperative care bundles to prevent surgical site infections are recommended.

### 4.3. Immediate Postoperative Phase

In the immediate postoperative phase of the management of a patient with SBO, which is performed by the surgeon, the anaesthesiologist and the nurse active temperature maintenance is mandatory and body temperature should be routinely measured with the goal to prevent hypothermia [27,72,73,74,75]. Oxygen saturation should be routinely measured to prevent hyposaturation and if needed oxygen therapy should be used. In the concept of the multidisciplinary management of those patients it is the responsibility of the whole team to achieve perioperative glycaemic control. For diabetic patients, local hospital protocols for diabetics undergoing surgery should be used while in patients at risk of developing insulin resistance (obese and elderly patients) and in surgeries lasting more than one hour, blood glucose levels higher than 180 mg/dL must be avoided. Thromboembolic prophylaxis consisting of compression stockings or intermittent compression and low-molecular weight heparin should be given according to the local hospital policy.

Regarding analgesia it is mandatory to implement opioid-sparing multimodal analgesia [81,82,83,84] and a restrictive fluid therapy protocol. Early mobilisation is one of the goals of the protocol in the immediate postoperative phase and the patients should sit up by two hours after surgery and should begin ambulation 8 h after surgery with respect to night time hours for sleeping [13,14,85,86,87,88,89].

In terms of feeding the patient should be kept nil per os while the withdrawal of the nasogastric tube should be assessed at 12 h after surgery and the removal of the urinary catheter, if it has been used, should be assessed 12 h after surgery.

### 4.4. First Postoperative Day

During the 1st postoperative day of the management of a patient with SBO, which is performed by the surgeon and the nurse perioperative glycaemic control is one of the key steps. For diabetic patients, local hospital protocols for diabetics undergoing surgery should be used while in patients at risk of developing insulin resistance (obese and elderly patients) and in surgeries lasting more than one hour, blood glucose levels higher than 180 mg/dL must be avoided [69,70,71]. Early mobilization is mandatory in the concept of the protocol and the patients should be fully ambulated in the 1st postoperative day while respiratory physiotherapy is a key element of the protocol [90,91,92,93,94].

Thromboembolic prophylaxis consisting of compression stockings or intermittent compression and low-molecular weight heparin should be given according to the local hospital policy.

Regarding the patients’ medications, antibiotic therapy should be given only in cases of bacterial translocation or abdominal cavity contamination. Broad spectrum antibiotics should be given according to the local hospital policy. Otherwise, the administration of antibiotic prophylaxis only is sufficient. Moreover, regarding pain management opioid-sparing analgesia must be used [55,56,59,95,96,97,98,99,100,101].

In order to easily mobilize the patient nasogastric tube, urinary catheter and epidural catheter removal should be considered. However, as the patients of the protocol are operated for bowel obstruction, while early nasogastric tube removal may be considered, due to the nature of the disease and high output this may not be feasible early in the postoperative period and the nasogastric tube may need to remain even till the second postoperative day. If the nasogastric tube is removed consider commencing a liquid diet or semisolid diet [70,102,103].

### 4.5. Second Postoperative Day

During the 2st postoperative day of the management of a patient with SBO, which is performed by the surgeon and the nurse perioperative glycaemic control is one of the key steps. For diabetic patients, local hospital protocols for diabetics undergoing surgery should be used while in patients at risk of developing insulin resistance (obese and elderly patients) and in surgeries lasting more than one hour, blood glucose levels higher than 180 mg/dL must be avoided [69,70,71]. Early mobilization is mandatory in the concept of the protocol and the patients should be fully ambulated in the 1st postoperative day while respiratory physiotherapy is a key element of the protocol [90,91,92,93,94]. Thromboembolic prophylaxis consisting of compression stockings or intermittent compression and low-molecular weight heparin should be given according to the local hospital policy. Moreover, regarding pain management opioid-sparing analgesia must be used per os.

In order to easily mobilize the patient nasogastric tube removal should be considered. However, as the patients of the protocol are operated for bowel obstruction, while early nasogastric tube removal may be considered, due to the nature of the disease and high output this may not be feasible early in the postoperative period and the nasogastric tube may need to remain even till the second postoperative day. If the nasogastric tube is removed, consider commencing a liquid diet or semisolid diet. Lastly, early discharge should be assessed according to discharge criteria for cases without intestinal resection.

### 4.6. Third Postoperative Day

During the 3rd postoperative day of the management of a patient with SBO, which is performed by the surgeon and the nurse, early feeding and early mobilization are key elements as well as respiratory physiotherapy and thromboprophylaxis. Early discharge should be assessed according to discharge criteria.

### 4.7. Discharge

Patient discharge from the hospital involves the surgeon, nurse and primary care. Regarding thromboprophylaxis, continued individualized thromboprophylaxis should be administrated according to risks while regarding antibiotic therapy it should be consider continuing antibiotic therapy in an outpatient setting in patient with indications. For the patient to be discharged the laboratory blood test should show at least a 50% decline in CRP prior to discharge. Follow-up is mandatory for all patients and follow-up after discharge at 24 h in an outpatient setting or via telephone should be done. Also, patients should be invited for a further follow-up visit according to local hospital policy and if necessary, home support with primary care physician should be coordinated.

Finally, the general discharge criteria are no complications that cannot be managed in an outpatient setting, return of regular bowel movements, no fever, pain controlled with oral analgesia and, importantly, acceptance by the patient.

**Figure 1:**
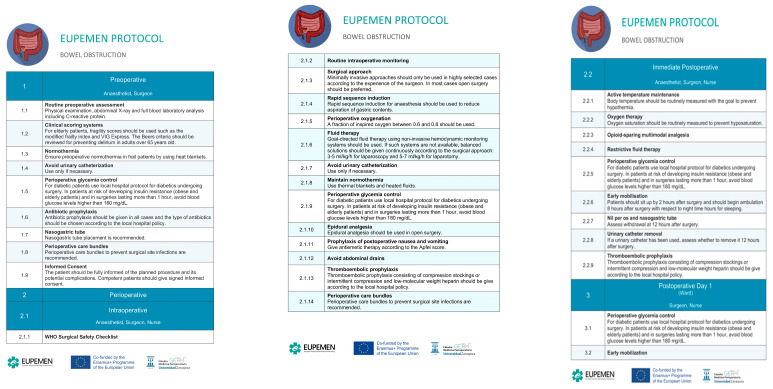

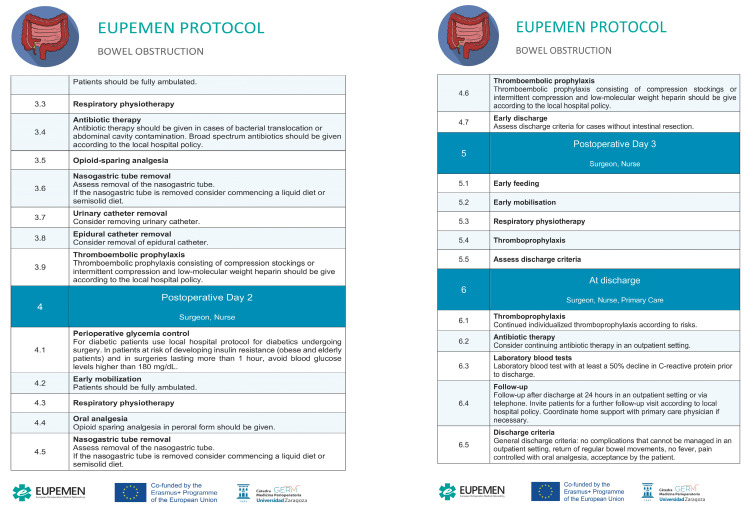
The EUPEMEN Protocol for Bowel Obstruction for the Preoperative, Intraoperative and Postoperative Period.

## 5. ERAS in Bowel Obstruction

The ERAS protocols have been proved to be effective in reducing postoperative complications, morbidity, length of hospital stay and overall cost in elective surgical procedures. While it seems more difficult to implement these protocols in emergency surgery, there are a few studies that have showed not only the feasibility of the protocols in bowel obstruction, both and mainly of the large intestine but also of the small intestine too, but also its advantages [104]. Specifically, the implementation of the ERAS protocol in patients undergoing emergency surgery for bowel obstruction has led to shorter hospital stay [104,105,106,107], decreased morbidity including pulmonary complications, paralytic ileus, surgical site infections [104,106], an increased recovery of gastrointestinal function including decreased time to passing flatus [104], less postoperative pain and better quality of life [104,106], a decreased inflammatory response including reduced CRP and procalcitonin values and also an increased efficacy of treatment in the ERAS group [106].

## 6. The EUPEMEN Project

The goal of the EUPEMEN project is to bring together the expertise and experience of national clinical champions who have previously helped to deliver major change programmes in their countries and to use them to spread these protocols in Europe. This main goal has been achieved with the next specifics objectives:

1. Preparation of an educational project (that included a teaching the teachers’ model);
2. Implementation in a significant number of European hospitals of the evidence-based protocols in a homogeneous and standardised way;
3. Collection of data about hospital stay, morbidity and mortality of European Surgical patients that once analysed through machine learning algorithm, will be of relevant interest to better know the surgical risk of an individual patient, hence to prevent perioperative complications.

The direct target groups the project aims are health professionals who are directly in charge of the care of surgical patients: surgeons, anaesthesiologists and nurses, as well as health professionals related to the interdisciplinary treatment of these patients: nutritionists, stoma-therapists, physiotherapists, rehabilitators, gastroenterologists, radiotherapists, oncologists, and pathologists. Moreover, as the effectiveness, as depicted by reduction of hospital stays and optimization of the use of other resources, is one of the advantages of these programs, health centres administrator, clinical managers and quality coordinators will also benefit from the project. Finally, due to the characteristics of enhanced recovery, primary care physicians play a very active role too. The indirect target groups of the project are the patients and their relatives and the patients’ associations, while the project’s stakeholders are local, regional, and national authorities and diseases associations.

The technical activities of the project were:

1. The preparation of the EUPEMEN Multimodal Rehabilitation manual with the protocols of 6 different modules:
  - Bariatric Surgery;
  - Oesophageal Surgery;
  - Gastric Cancer Surgery;
  - Colon Surgery;
  - Hepatobiliary Surgery;
  - Urgent abdominal surgery, including appendectomy and small bowel obstruction (SBO).
2. The development of the EUPEMEN online platform (https://eupemen.eu/, accessed on 24 April 2023): to host the e-learning training course and a collaborative area to improve and to participate in the protocols;
3. The training of the trainers to teach the future teachers the different protocol to be able to teach them in the different hospitals;
4. The dissemination of the results in 5 Multiplier events, one per partner, to promote the protocols;
5. The organization of 4 transnational meetings, one per country;
6. The translation into English of the Recovery Intensification for optimal Care in Adult’s surgery—RICA from the Spanish de Recuperación Intensificada en Cirugía del Adulto (RICA).

The results of the project were the development of the EUPEMEN Protocols Training Programme for health professionals, the training of 200 multidisciplinary professionals in all the direct target groups involved in perioperative procedure from each partner in one local forum with 40 participants. Furthermore, the implementation of the protocols in, at least, 5 hospitals in Europe and the creation of a professional network with capacity to train stakeholders in hospitals, and to audit the trainers to guarantee the correct implementation of the programme. Long-term effect and impact of the project will be to decrease the secondary effects after surgery for patients, consequently, with a faster patient recovery, to reduce morbidity and mortality caused after surgical operations and to reduce the length of stay in the hospital and, consequently, save money for the public health system and to have more free beds for other new requested patients.

## 7. Discussion

The main objective of the project is the uniform, consistent, consensual and multi-centre implementation of the program of perioperative medicine based on the evidence resulting from the clinical pathway of Recovery Intensification for optimal Care in Adult’s surgery (RICA), published by the Ministry of Health, Social Services and Equality and the Aragon Health Sciences Institute, in the hospital centres at a European level, as the implementation of the program in hospitals will mean an important decrease in perioperative complications (morbidity and mortality) in patients included in the program, as well as a shortening of global hospital stays, an improvement in the efficiency of professionals, the inclusion of patients and caregivers in the making of decisions concerning processes, a better and earlier reincorporation of them in their family and social/work environment and an overall improvement of the care given; all of which is related to an overall decrease in the cost per process and resulting in safer processes. The two Intellectual Outputs that were developed were a training manual for the implementation and correct execution of the protocols and a teaching and learning platform completed through the collaboration and cooperation of the participants. The advantages of the project were that these intellectual outputs are innovative and have a transnational added value, as they will be elaborated taking into account the particularities of the different health systems of the participating countries, which will help to elaborate valid protocols in the different countries of the EU, the researchers involved in the project are professionals with experience in research and innovation, and also the innovation of the proposal to modify clinical practice by making it safer and promoting teamwork through the creation of multidisciplinary clinical units that will create synergies that will demonstrate clinical talent and excellence. The evaluation of the implementation results is proposed as a secondary objective by means of the analysis of established indicators and comparing the previously known clinical results with those from the new program, both in the short and long term.

## Data Availability

Data is contained within the webpage. The data presented in this study are available in [https://eupemen.eu/, accessed on 24 April 2023].
